# Managing the screen-viewing behaviours of children aged 5–6 years: a qualitative analysis of parental strategies

**DOI:** 10.1136/bmjopen-2015-010355

**Published:** 2016-03-01

**Authors:** R Jago, J Zahra, M J Edwards, J M Kesten, E Solomon-Moore, J L Thompson, S J Sebire

**Affiliations:** 1 Centre for Exercise, Nutrition & Health Sciences, School for Policy Studies, University of Bristol, Bristol, UK; 2 School of Social and Community Medicine, University of Bristol, Bristol, UK; 3 School of Sport, Exercise and Rehabilitation Sciences, University of Birmingham, Birmingham, UK

**Keywords:** Screen-viewing, Children, Interview, Parent

## Abstract

**Objectives:**

The present study used qualitative methods to: (1) examine the strategies that were used by parents of children aged 5–6 years to manage screen viewing; (2) identify key factors that affect the implementation of the strategies and (3) develop suggestions for future intervention content.

**Design:**

Telephone interviews were conducted with parents of children aged 5–6 years participating in a larger study. Interviews were transcribed verbatim and analysed using an inductive and deductive content analysis. Coding and theme generation was iterative and refined throughout.

**Setting:**

Parents were recruited through 57 primary schools located in the greater Bristol area (UK).

**Participants:**

53 parents of children aged 5–6 years.

**Results:**

Parents reported that for many children, screen viewing was a highly desirable behaviour that was difficult to manage, and that parents used the provision of screen viewing as a tool for reward and/or punishment. Parents managed screen viewing by setting limits in relation to daily events such as meals, before and after school, and bedtime. Screen-viewing rules were often altered depending on parental preferences and tasks. Inconsistent messaging within and between parents represented a source of conflict at times. Potential strategies to facilitate reducing screen viewing were identified, including setting screen-viewing limits in relation to specific events, collaborative rule setting, monitoring that involves mothers, fathers and the child, developing a family-specific set of alternative activities to screen viewing and developing a child's ability to self-monitor their own screen viewing.

**Conclusions:**

Managing screen viewing is a challenge for many parents and can often cause tension in the home. The data presented in this paper provide key suggestions of new approaches that could be incorporated into behaviour change programmes to reduce child screen viewing.

**Strengths and limitations of this study**

- The study provides new information on an important but relatively underexplored public health topic; the strategies that parents use to manage their young child's screen viewing.
- Recruitment of participants via sampling within an existing cohort facilitated identification of children from a range of different socioeconomic groups with varying levels of physical activity.
- Sampling from within a larger survey also provided broader contextual information about the levels of screen viewing of the child being discussed by the parent.
- The study is limited by the small number of fathers who took part in the interviews.

## Introduction

Screen viewing (ie, watching TV, using computers, tablets, smartphones and playing on game consoles) has been associated with higher levels of obesity,^1^ metabolic risk,^2^ decreased psychological well-being^3^ ^4^ and lower scores in national examinations.^5^ A number of studies have shown that large proportions of children and adolescents exceed the American Academy of Paediatrics’ recommendation that children's total screen-viewing time should not exceed 2 h/day.^6–11^ TV viewing, the most extensively studied form of screen viewing, tracks from childhood to adulthood.^12^ Many preschool-aged children engage in regular screen viewing^13^ and by the start of primary (elementary) school screen viewing is well established among children.^1^ ^14^ As such, finding ways to minimise screen viewing among children aged 5–6 years is likely to be important for current and future health.

Parents are important influences on children's screen-viewing behaviours. A number of previous studies have shown that there is strong evidence of an association between parent and child screen time.^9^ ^15^ A growing body of work shows that parental rule setting is associated with lower levels of screen viewing.^16–18^ These studies suggest that the actions a parent takes are likely to affect children's screen viewing, and that parents could play important roles in screen-viewing behaviour change interventions. To date, the majority of research in this area has utilised quantitative approaches. Insight gained from qualitative research could be important in developing a detailed understanding of the experiences of parents, particularly in relation to the barriers and facilitators to managing their children's screen-viewing behaviours. These data may help identify ways in which to help parents regulate child screen-viewing time.

The UK Medical Research Council's (MRC) guidance on the development of complex interventions^19^ states that effectiveness is likely to be enhanced if intervention content is developed in a step-wise process. A key component in this process is identifying strategies that are used by members of the target group that could be translated more widely, as well as identifying potential new strategies. This study aimed to: (1) examine the strategies used by parents of children aged 5–6 years to manage screen viewing; (2) identify key factors that might affect the implementation of these strategies and (3) develop suggestions for future intervention content.

## Methods

Data presented here are from the B-ProAct1v baseline study which included the assessment of parent and child screen-time; the methods of this study have been reported previously.^15^ ^20^ Briefly, the B-ProAct1v baseline study included 1267 children aged 5–6 years and at least one of their parents, who were recruited from 57 schools within the wider Bristol area. All children, and at least one parent, wore an accelerometer for up to 5 days and were included in the analysis if they provided at least 3 days of valid data, where a valid day was defined as the provision of at least 500 min of data.^21^ The mean minutes of moderate-to-vigorous intensity physical activity (MVPA) and sedentary time across the valid days were then calculated for children using the Evenson accelerometer thresholds.^22^ Parents also completed a questionnaire in which they recorded the amount of time that they and their child spent using TV, computer/laptop and consoles, on weekdays and weekends (<1, 1–2, 2–3, 3–4, 4 h or more). The questionnaire used to assess screen viewing has been previously used with children and parents of children aged 10–11 years 17 23 24 and was chosen because the assessment of TV viewing using parental response to a single question has been shown to correlate moderately (r=0.60) with 10 days of TV diaries among young children.^25^

From the larger sample, a subsample of parents was recruited to participate in in-depth interviews. To ensure variability in the experiences of the interview subsample, parents were purposively sampled based on ‘low’, ‘medium’ and ‘high’ thirds of child MVPA and thirds of deprivation which was estimated from the Index of Multiple Deprivation^26^ for the home postcode and treated as an indicator of social economic position (SEP). This created nine groups and a random stratified sampling process was then conducted to invite parents from within these groups to take part in a telephone interview. In total, 274 parents were invited to participate in the interviews and 53 interviews were conducted. The study was approved by the School for Policy Studies Ethics and Research Committee at the University of Bristol. Written informed consent was obtained from all participants, and participants received a £10 gift voucher in recognition of their time.

Data were collected via semistructured interviews. Telephone interviews were used as our previous research confirms that this is an effective means of collecting qualitative data from parents,^27–29^ and evidence suggests that telephone interviews can provide as reliable information as face-to-face interviews.^30^ The interview guide was iteratively developed by the project team and refined during the data collection period. Interviews examined ways in which parents managed their child's screen time and any specific issues they encountered in this process. Specifically, questions related to managing screen-viewing time including the tactics or strategies that they used and challenges experienced, encouragement and discouragement of screen viewing, parental messages/communication relating to child screen viewing and message consistency, rules and rule enforcement and disagreements with children regarding screen viewing. Interviewers checked understanding during the course of the interviews with participants. All interviews were digitally recorded, transcribed verbatim and the transcripts were reviewed for accuracy by the researcher who conducted the interview. To provide contextual information about each family, we have indicated after each quote the mean minutes of child MVPA, mean sedentary minutes, weekday hours of TV viewing, child gender and socioeconomic group (low, medium or high socioeconomic position).

### Analysis

Content analysis was performed using inductive and deductive approaches.^31^ Initially, all transcripts were read multiple times by at least two researchers. An initial coding frame was developed and applied to the data based on pre-existing ideas pertaining to the existence of screen-viewing management strategies.^16^ ^17^ ^32–35^ The coding frame was refined during this process to allow for the inductive emergence of additional themes with weekly discussion among the project team to ensure accuracy and consistency. Once transcripts had been coded, a second researcher checked the codes and identified any discrepancies, which were discussed and agreement achieved. Data were entered into and coded within NVivo (V.10, QSR, Southport, UK). Hierarchies of categories were created and summarised, and brief summaries and representative quotes for each category are reported.

## Results

There were seven themes that emerged from the data pertaining to the strategies that parents used to manage their child's screen viewing. These strategies were: (1) using screen viewing as reward and punishment; (2) limit setting in relation to daily events; (3) context-specific limit setting and when limits were relaxed; (4) offering alternatives; (5) consistency; (6) negotiation and compromise and (7) child self-regulation. An overview of each theme and illustrative quotes are presented below.

### Screen viewing as reward and punishment

Parents reported using screen time as a reward for good behaviour. The restriction of screen time was used as a punishment, which often resulted in conflict. There was also evidence that as screen viewing was a behaviour desired by children, limiting screen viewing was best achieved by negotiating using desirable alternative activities.

Not having screen time is kind of a consequence for misbehaviour. [Interview 13; Mother, Girl, Weekend TV <1 h, Weekday <1 h, 69 MVPA minutes, 406 Sedentary minutes High SEP]

He has to kind of earn it [screen-viewing] if you see what I mean. [Interview 30; Mother, Boy, Weekend 2–3 h, Weekday 1–2 h, 70 MVPA minutes, 380 Sedentary minutes Low SEP]

At the moment he's really enjoying a famous five story that we're reading to him, so that's a good incentive; ‘if you turn it off now, you can have two chapters’. [Interview 37; Mother, Boy, Weekend 1–2 h, Weekday 1–2 h, 89 MVPA minutes, 323 Sedentary minutes High SEP]

### Limit setting in relation to daily events

Numerous parents reported simple limit setting in relation to the child's usual schedule, such as not allowing TV before school or during meal times, and prioritising homework over screen time.

We are not concerned but we keep an eye on it, so whilst he would wake up in the mornings and stick the TV on, if it is a school day, no. [Interview 22; Mother, Boy, Weekend 2–3 h, Weekday 1–2 h, 71 MVPA minutes, 455 Sedentary minutes, High SEP]

And homework as well because I like to … like 7 o'clock I make sure TV's off so it's all quiet and they all do their reading before bedtime. [Interview 15; Mother, Girl, Weekend 3–4 h, Weekday <1 h, 51 MVPA minutes, 354 Sedentary minutes, Medium SEP]

Some parents thought that their children could use timing devices to manage their own screen time, and that digital TV recorders (eg, Sky Plus, TiVo, etc) were helpful in encouraging children to be selective over their TV time.

So they can see it and they turn the timer on themselves. You have it on the table in front of the television and so they say “right you have got fifteen minutes of Harry Potter.” [Interview 2; Mother, Boy, Weekend 2–3 h, Weekday 1–2 h, 41 MVPA minutes, 352 Sedentary minutes, High SEP]

### Context-specific limit setting and when limits are relaxed

Many parents reported that screen-viewing limit setting and rules were circumstantial and that they were more willing to allow screen time when they were busy. Some parents suggested that household or family tasks, such as cooking dinner, or wanting time where they were not directly supervising their child, impacted their willingness to allow screen time.

[I allow] screen viewing, yes. If I want a lie in, in the morning. [Interview 49; Mother, Boy, Weekend 2–3 h, Weekday <1 h, 85 MVPA minutes, 263 Sedentary minutes, Medium SEP]

Like if I’m cooking tea or something like that, then I don't mind them because obviously I’m getting on doing something and they're quiet. [Interview 25; Mother, Girl, Weekend 1–2 h, Weekday <1 h, 71 MVPA minutes, 352 Sedentary minutes, Low SEP]

There was a consistent pattern whereby rules for screen time were often relaxed when parents felt that their child needed additional quiet time after a difficult day or week at school.

It could be she's had a really busy week at school and…everything's got to the point, sometimes that she needs a bit more of a break [resulting in more screen-time]. [Interview 47; Mother, Girl, Weekend 1–2 h, Weekday <1 h, 67 MVPA minutes, 390 Sedentary minutes, High SEP]

I sort of believe that, you know, they deserve a little bit of chill out time as well, I think especially when they've been at school all day, you know, and I think he comes home and he's tired. [Interview 16; Mother, Boy, Weekend 2–3 h, Weekday 1–2 h, 70 MVPA minutes, 450 Sedentary minutes, High SEP]

### Offering alternatives

The majority of parents reported offering alternatives to screen viewing as part of their screen-viewing management strategy and this strategy was often related to theme 1 which focused on screen viewing as a reward. Common alternative activities included playing with toys, physical activity, reading, cooking, board games and arts and crafts. The process of alternatives being offered varied between families, with some parents stating that children would happily choose the behaviour they wish to perform instead of a screen behaviour, while other parents described having to suggest or ‘stage’ alternatives.

I might say ‘that's enough of that Wii (Games Console), I'm turning it off, go and find something that doesn't involve a screen’ because they might then say ‘can I go on the sort of iPad’ or something?’ and I'll say, ‘No, it has to be something that doesn't involve any kind of computer technology,’ and they'll go off and do their art or something. [Interview 12; Mother, Boy, Weekend >4 h, Weekday 1–2 h, 76 MVPA minutes, 419 Sedentary minutes, Medium SEP]

I always try to encourage her to do something different like painting, colouring or something like this or we would go to park or we go shopping. [Interview 32; Mother, Girl, Weekend 3–4 h, Weekday 1–2 h, 112 MVPA minutes, 326 Sedentary minutes, Medium SEP]

Sometimes I have to stage things, like I'll get the Lego out on the table and they'll just be naturally drawn to it. [Interview 48; Mother, Girl, Weekend 2–3 h, Weekday <1 h, 41 MVPA minutes, 371 Sedentary minutes, Medium SEP]

### Consistency between parents

A number of participants talked about the importance of consistency between parents in managing child screen time; in some families consistency naturally occurred, whereas in others, inconsistency in relation to screen viewing was a source of frustration.

I suppose his mum [and] myself, we do try and be consistent actually. We do, without having sat down and one of these conversations between the two of us sort of thing he just sort of has worked out, we have come to realise that whilst he will try and play us off against each other, “oh mum says it is fine and dad says”, you know he knows which one of us to try and tackle…first. [Interview 22; Father, Boy, Weekend 2–3 h, Weekday 1–2 h, 71 MVPA minutes, 455 Sedentary minutes, High SEP]

We have had a few disagreements in the past, I am sure there will be more. Yes I am quite strict with it but sometimes [his Father] doesn't see the harm and…he is not seeing the fall out because they are tired or because they are more wound up instead of chilled out and calmed down before bed. [Interview 18; Mother, Boy, Weekend 1–2 h, Weekday <1 h, 115 MVPA minutes, 358 Sedentary minutes, Medium SEP]

There seemed to be a tendency to refer to fathers as particularly influential in relation to screen time at the weekend, and that fathers were often more relaxed about managing screen-viewing behaviours than mothers.

No, their dad has much more [influence on screen-viewing]…Because he likes it himself. I am not bothered either way. [Interview 18; Mother, Boy, Weekend 1–2 h, Weekday <1 h, 115 MVPA minutes, 358 Sedentary minutes, Medium SEP]

I probably have more influence in the week and then my husband maybe has more influence on the weekends… [Interview 33; Mother, Boy, Weekend 1–2 h, Weekday <1 h, 38 MVPA minutes, 284 Sedentary minutes, High SEP]

He's (Father) probably more relaxed about screen-viewing and that's probably more to do with his upbringing. [Interview 17; Mother, Boy, Weekend 1–2 h, Weekday 1–2 h, 82 MVPA minutes, 341 Sedentary minutes, Low SEP]

### Negotiation and compromise

Parents talked about negotiating screen time with their children to minimise conflict, and specifically discussing with the child when to finish screen viewing at a convenient point in the programme or game. This latter strategy was facilitated by the use of TV-recording devices which prevented conflict about the child missing a particular programme.

For example, last night, Sunday night, they have done everything we need to do, it is getting towards bedtime, we have turned the telly on at like just gone seven o'clock and the film finishes at half past seven. I am wanting to put them to bed; they are upset because they are watching a little twenty minutes of Harry Potter before they go to bed. Every day they want to do that, so I am thinking ‘well it is not that late’ and then I let them watch fifteen minutes. [Interview 2; Mother, Boy, Weekend 2–3 h, Weekday 1–2 h, 41 MVPA minutes, 352 Sedentary minutes, High SEP]

If [child name] is particularly enjoying something it's very easy to say to her, you know, ‘it is bedtime, will you stop watching this now? Mummy will record the rest of it, and you can watch it another time’. [Interview 17; Mother, Girl, Weekend 1–2 h, Weekday 1–2 h, 82 MVPA minutes, 341 Sedentary minutes, Low SEP]

### Child self-regulation

A number of parents reported that their children could regulate their screen viewing, and that this was something that the parents encouraged.

We'd like him to become better at policing it himself so he, he is quite good at saying ‘I don't want to watch this programme, I'll turn it off’. But yes, if he could, he'd play on the Wii all day I think. [Interview 37; Mother, Boy, Weekend 1–2 h, Weekday 1–2 h, 89 MVPA minutes, 323 Sedentary minutes, High SEP]

She likes watching Cbeebies (BBC Children's TV channel), but she's actually much better herself at policing that. If she doesn't want to watch one programme she will just turn it off or walk away. [Interview 37; Mother, Boy, Weekend 1–2 h, Weekday 1–2 h, 89 MVPA minutes, 323 Sedentary minutes, High SEP]

Parents also suggested that children self-regulate their screen viewing and that when they get bored of screen behaviours they find something else to do.

He will watch a programme but he tends to, after about half an hour, get a bit itchy feet. [Interview 38; Mother, Boy, Weekend 2–3 h, Weekday 1–2 h, 80 MVPA minutes, 411 Sedentary minutes, Low SEP]

## Discussion

The data presented in this paper highlight a number of strategies used by parents to limit the screen time of their child aged 5–6 years, and the associated factors within the daily lives of families that are likely to affect the outcome of such strategies. An underlying theme of the data is the central role that screen viewing has in children's lives and the complexity that families face in managing children's screen time. The interviews give numerous examples of parents struggling to find a balance in managing a behaviour that they and their children enjoy, and which parents can benefit from, by giving them time to perform other domestic tasks such as cooking. These findings are therefore consistent with previous research which has suggested that the TV is often used as a ‘babysitter’ providing opportunities for parents to complete other tasks.^36^ As a result, screen time is often a reward for good behaviour and removal of screen time is a punishment. The appeal of screen time was further typified by the many instances in which parents reported having to negotiate conflict around limiting screen. This reward and punishment role for screen viewing is in many ways comparable to the dietary literature, which has shown that encouraging children to eat particular foods, such as vegetables, before they can eat a dessert increases their preference for the rewarded food.^37^ As such, using screen viewing as a reward and removal as a punishment, has the potential to reinforce the perception within the home of screen time being a particularly appealing behaviour. Collectively, these findings demonstrate an important need to find and develop ways to help families manage their child's screen time.

Parents reported a number of strategies to manage screen time, such as limiting screen time to set periods of the day, not eating meals in front of the TV and completing homework before screen time. These strategies match a number of strategies reported in the literature,^16^ ^29^ ^35^ but the data also suggest the importance of parents having a range of alternative activities that they can direct children to engage in. Parents also reported the use of a number of preplanned and tested negotiation and communication strategies to prevent or minimise conflict. An underlying theme that emerged from the data was the sedentary nature of screen-viewing alternatives utilised by parents. This could relate to parents perceiving children to need rest after the school day, potentially due to them being in their first year of primary school. No parents suggested other activities, such as helping them with tasks that they were trying to complete while the child watched TV, such as cooking or preparing the table for dinner. As such, a key area of future work is to develop a broader range of alternative activities in which parents can encourage their children to engage. Combining these activities with new means of improving parental communication and negotiation skills are likely to be important for managing and reducing child screen time.

The findings suggested that understanding the context within which screen viewing is occurring is critical in facilitating strategies to minimise screen time. The data clearly suggest that knowing when to intervene, and when not to challenge screen time, is important for many parents. Furthermore, interviews highlight the importance of developing adaptable strategies to minimise screen time in a range of different circumstances so that parents have a preidentified set of solutions. Parents reported that their actions, such as whether TV was allowed in the morning, were different for school days and non-school days (weekends or school holidays). In the UK, schools are open for 190 days/year,^38^ therefore there are 174 non-school days per year. Moreover, as previous research has suggested that physical activity and screen-viewing patterns on a Friday often differ from other weekdays, and are considered part of the weekend, there is a need to focus on managing screen viewing on weekday and weekends.^39^

The negotiation and compromise theme suggested that conflict caused by the removal of screen time is likely to be reduced if screen-viewing rules are set in conjunction with the children. This finding is consistent with our previous work, which has shown that collaborative rule setting is something that children desire and is associated with lower screen viewing among children aged 6–8 years.^34^ ^40^ Furthermore, O'Connor *et al*^35^ has suggested that collaborative rule setting in relation to screen viewing is a parenting practice that warrants further examination; we are unaware of any study that has tested the potential of this parenting approach in an intervention setting. As such, testing the potential of this approach of working with parents to collaboratively set screen-viewing rules is warranted.

An overall impression of the data is that many parents appear to be giving mixed, highly nuanced information to their children in relation to screen viewing. For example, for some parents screening-viewing was not acceptable in the morning, unless the parent desired extra time in bed. Another example was that screen viewing is not something that children should engage in a lot, unless they have been well behaved, in which case it is acceptable. Mixed messages from parents have been identified as a consistent source of tension in families and many parenting programmes focus on the establishment of consistent messages in general parent–child communication as a means of minimising conflict.^41^ ^42^ Thus, a challenge for researchers is to identify how consistent messages can be developed, and how these messages can take account of the different contexts in which parents change their management approach.

The evidence presented in this paper highlights the potentially important role that fathers play in the management of child screen time. Previous research has shown that screen-viewing rules are associated with less TV viewing when supported by both parents as opposed to just one, and these data appear to confirm this finding while highlighting the important role of fathers.^17^ Involving fathers in behaviour change programmes and research is challenging,^43^ and although there have been a few successful studies in this area,^44^ ^45^ many studies have reported that fathers are a group that are harder to recruit and engage than mothers. As such, there is an important need to: (1) recruit fathers to research projects and (2) work with fathers to find ways to help them manage their child's screen time. Another issue which was not greatly discussed in these interviews, but for which the research team felt was in the background, was how screen viewing is managed for children who have separated parents. In this instance, finding ways in which parents can utilise similar strategies would, although undoubtedly hard to do, be helpful to children and parents.

The key strategies that could be used to limit the screen-viewing behaviours of young children that were identified from the data presented in this paper and how they could be addressed are summarised in table 1. These suggestions imply that reducing screen viewing is complicated, and helping parents to navigate the complex issues around screen time may require considerable effort. This could be achieved via the use of in-depth parenting programmes, which have been highlighted as intervention approaches that have potential for helping parents to manage screen time.^46–49^ Parenting programmes are, however, time and resource intensive^46–49^ and as such the challenge for public health researchers is to develop such interventions in a cost-effective manner.

**Table 1:** Themes identified from the analysis of parent interviews and potential implications for screen-viewing behaviour change interventions

Theme	Implication for screen-viewing behaviour change interventions
Screen viewing seen as a reward and screen-viewing removal a punishment	Need to change perception of screen viewing in the home Develop strategies to downplay importance of screen viewing Appeal to parents’ interests/values in reducing screen viewing such as enhancing quality family time or balancing time on high-tech vs low-tech activities
Setting screen-viewing limits in relation to daily events	Simple strategies include No screen viewing before school No screen viewing during meals No screen viewing until after key tasks such as homework Provide timers to encourage children to self-monitor Use digital recording devices to prioritise viewing options
Context-specific limit setting	Identify when screen viewing is occurring Identify in advance when and how screen-viewing rules will be adapted/relaxed Identify how to accommodate screen viewing on ‘non-normal’ days Set screen-viewing rules as a family
Offering screen-viewing alternatives	Have preset list of ‘go to activities’ Stage non-screen-viewing activities to pique interest
Consistency between parents	Engage both parents in rule making and enforcement Ensure consistency when one or two parents are at home Work collaboratively for consistency for shared custody
Negotiation and compromise	Engage children in screen-viewing rule-setting process
Child policing/self-regulation	Build child skills to self-monitor screen viewing Praise child engagement in alternative behaviours

### Strengths and limitations

The major strength of this study is the depth of information that was available about the amount of screen viewing in which the children who the parents were talking about engaged in. This was possible as the participants were purposively sampled from a large cross-sectional study to provide a range of physical activity patterns from across the socioeconomic spectrum. Moreover, 53 participants is a relatively large sample for qualitative research, and saturation had been met in the interviews. However, the data are limited to the participants who were interviewed, and the findings need to be extrapolated to the wider population with caution. In particular, few fathers were interviewed and further research to understand the role of fathers in the management of screen viewing is warranted.

## Conclusion

Screen viewing is a desirable behaviour for many children aged 5–6 years and is complexly entwined in the lives of families with young children. Managing screen viewing is a challenge for many families and is often a source of inconsistent messaging within and between parents and conflict. Potential strategies to facilitate improved screen-viewing behaviours that were informed by interviews with parents include setting screen-viewing limits in relation to specific events, collaborative rule setting, monitoring that involves mothers, fathers and the child, developing a family-specific set of alternative activities to screen viewing and developing children's awareness of screen viewing time in order to encourage and improve self-monitoring.
